# Real-world Treatment Patterns and Outcomes in HR+/HER2+ Metastatic Breast Cancer Patients: A National Cancer Database Analysis

**DOI:** 10.1038/s41598-019-54402-9

**Published:** 2019-12-02

**Authors:** Abby B. Statler, Brian P. Hobbs, Wei Wei, Annie Gupta, Cassann N. Blake, Zeina A. Nahleh

**Affiliations:** 1 Cancer Biostatistics, Taussig Cancer Institute, Cleveland Clinic, Cleveland, OH USA; 2 Quantitative Health Sciences, Taussig Cancer Institute, Cleveland Clinic, Cleveland, OH USA; 3 Department of Internal Medicine, Cleveland Clinic Florida, Weston, FL USA; 4 Department of General Surgery, Cleveland Clinic Florida, Weston, FL USA; 5 Department of Hematology/Oncology, Maroone Cancer Center, Cleveland Clinic Florida, Weston, FL USA

**Keywords:** Targeted therapies, Breast cancer

## Abstract

Treatment patterns and outcomes are unclear for metastatic breast cancer (MBC) patients diagnosed with hormone receptor-positive (HR+), human epidermal growth factor 2-positive (HER2+) disease. This study aimed to: (1) examine the utilization of first-line therapy among HR+/HER2+/MBC patients and (2) compare overall survival (OS) between the identified regimens. We analyzed National Cancer Database patients (HR+/HER2+/MBC) who were treated between 2010 and 2015. Multivariable logistic and Cox regression were used to: (1) identify independent predictors of treatment receipt and (2) determine significant prognostic factors for OS. Kaplan-Meier method and log-rank test were used to estimate and evaluate OS, respectively. Propensity scores were added to all multivariate OS models, thereby accounting for bias in treatment receipt. Of 6,234 patients analyzed, 3770 (60.5%) received hormonal therapy and 2464 (39.5%) received chemotherapy. Receipt of hormonal therapy was associated with older age, grade 1/grade 2 disease, no visceral involvement, higher comorbidity scores, and being white. Multivariate analysis suggest patients receiving hormonal therapy + anti-HER2 experienced improved OS, when compared to chemotherapy + anti-HER2 (HR: 0.74, p = 0.004). Overall, the cohort receiving hormonal therapy + anti-HER2 reported the highest 5-year OS (hormonal + anti-HER2: 47.5% vs. chemotherapy + anti-HER2: 39.8% vs. hormonal: 38.5% vs. chemotherapy: 36.3%, p < 0.001). Our findings suggest de-escalated therapy may be the preferred and potentially more effective care path for HR+/HER2+/MBC patients, signaling a need for randomized studies.

## Introduction

Despite successful human epidermal growth factor 2-positive (HER2+) directed therapies, many patients with advanced HER2+ breast cancer will eventually develop treatment resistance and succumb to their disease. There are several treatment options for patients with advanced hormone receptor positive (HR+) and HER2+ breast cancer^1^. However, there is an ongoing debate in clinical practice regarding the best first-line treatment approach for a newly diagnosed HR+/HER2+ metastatic breast cancer (MBC). Recently updated ASCO guidelines^1^ recommend HER2-targeted therapy combinations for first-line treatment of HR+/HER2 MBC, except for highly selected patients with estrogen receptor–positive (ER+) or progesterone receptor–positive (PR+) and HER2-positive disease, for whom endocrine/hormonal therapy (i.e. hormonal therapy) may be used alone. While the National Comprehensive Cancer Network (NCCN) guidelines^2^, which were updated in November 2018, recommend either hormonal therapy with or without HER2 targeted therapy or chemotherapy with HER2 targeted therapy.

Although the survival benefit of chemotherapy and anti-HER2 combination therapy has been established^3,4^; trends towards de-escalation are beginning to emerge, particularly as several novel anti-HER2 agents in advanced breast cancer are developing^5–7^. Trends with respect to utilization and clinical outcomes for HR+/HER2+ patients have not been described in the first-line setting for hormonal therapy plus anti-HER2 when compared to chemotherapy plus anti-HER2 therapy^8,9^. We therefore aimed to: (1) examine current practices in the utilization of hormonal therapy vs chemotherapy for the first-line treatment of HR+/HER2+ metastatic breast cancer, and (2) explore variations in overall survival among real-world patients.

## Methods

### Data sources

This study used the National Cancer Database (NCDB), a hospital-based registry that is jointly sponsored by the American Cancer Society (ACS) and the American College of Surgeons. This source includes data from more than 1,500 Commission on Cancer (CoC)-accredited facilities, representing approximately 70% of all malignancies in the United States, comprising more than 29 million unique cancer diagnoses^10,11^. The facilities participating in this registry seek accreditation by CoC, which requires an annual 90% follow-up rate for all eligible patients diagnosed within 5 years. All participating programs collect data prospectively and are required to adhere to best recommended practices for accurate documentation/transmission of known patient-level prognostic data as well as ascertain treatment outcomes^12^.

### Analysis population

**Figure 1:**
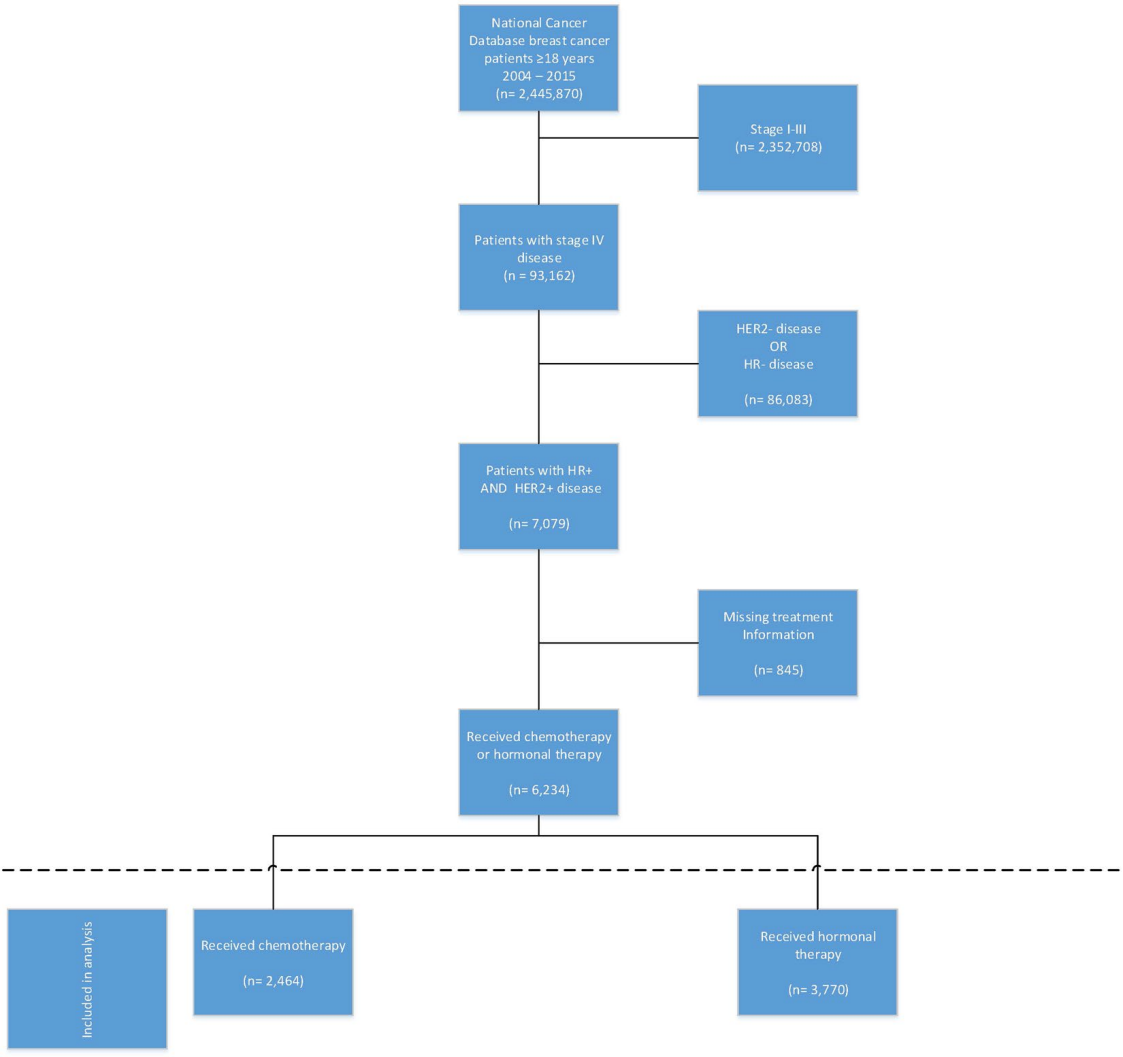
CONSORT diagram. HER, human epidermal growth factor; HR, hormone receptor.

This analysis included patients 18 years or older diagnosed with stage IV (defined as metastatic to a distant site, M1 per American Joint Committee on Cancer TNM Staging Criteria), hormone receptor positive (ER+ and/or PR+) and HER2+ breast cancer who received treatment between 2010 and 2015. Patients treated during this 5-year period were included because: (1) HER2 status and anti-HER2 treatment reporting became a requirement in 2010 and (2) 2015 was the last year in the dataset we requested. Patients diagnosed with non-metastatic (stages I-III), HR-, or HER2- disease were excluded (Fig. 1).

### Variables

The following variables were extracted for all patients meeting the inclusion criteria: age (<50 years, 50–70 years, >70 years), sex, race/ethnicity (white non-Hispanics, African American (AA), Hispanic/Latinos, Asian, others, unknown), Charlson/Deyo comorbidity score (0, 1, 2, ≥3 comorbid conditions), insurance status (not insured, private insurance, government insurance (Medicaid, Medicare, Tricare, Veterans Affairs), and unknown), median community income level (<$38,000, $38,000–$62,999, ≥$63,000), facility type (community cancer center, comprehensive community cancer center, academic/research cancer center, integrated network), distance to center (<4 miles, 4–8.9 miles, 9–17.9 miles, and ≥18 miles) year of diagnosis, grade (grade 1/grade 2 (well/moderately differentiated), grade 3/grade 4 (poorly differentiated/un-differentiated/anaplastic), unknown), visceral involvement (lung or liver metastases), number of metastatic sites (1, 2–3, >3), and treatment (chemotherapy, hormonal therapy, anti-HER2 therapy).

### Statistical analysis

Descriptive statistics were used to summarize the patterns of care regarding the use of hormonal therapy or chemotherapy. Chi-squared tests were performed to compare patient and contextual characteristics between the identified treatment patters. Multivariable logistic regression analyses [backward elimination] were conducted to identify independent predictors of treatment receipt (chemotherapy vs. hormonal therapy). Multivariate Cox proportional hazards regression was used to: (1) identify significant prognostic factors for OS [backward elimination] and (2) determine the effects of treatment choice on survival, after controlling for prognostic factors. Propensity scores were added to the multivariate overall survival models, thereby accounting for bias in treatment receipt. Variables contributing to the propensity of receiving hormonal therapy or hormonal therapy + anti-HER2 included: age, race, grade of disease, year of diagnosis, visceral involvement, and comorbidities. The distribution of the propensity scores generated for each model are shown in the Appendix, Figs. A1 and A2. Kaplan-Meier method was used to estimate distributions of overall survival, which were compared among patient cohorts using the log-rank test. Median follow-up time was calculated for all alive patients. All tests were two-sided, and p-values of 0.05 or less were considered to be statistically significant. Analyses were performed using SAS Studio 3.7 and R version 3.4.2.

### Ethics statement

The NCDB provides a de-identified Participant Use Data File, which is compliant with the Health Insurance Portability and Accountability Act. The Cleveland Clinic Institutional Review Board approved this study (FLA 18–087) and waived the informed consent requirement because the data are de-identified.

## Results

### Population characteristics

Of the 6,234 patients diagnosed with stage IV HR+/HER2+ breast cancer, the majority were 50 years or older (n = 4602 [73.8%]), female (n = 6141 [98.5%]), white (n = 4491 [72.0%]), had grade 3/grade 4 disease (n = 2912 [46.7%]), visceral involvement (n = 3276 [52.6%]), 1 metastatic site (n = 4003 [64.2%]), and reported no comorbidities (n = 5161 [82.8%]). Year of diagnosis and distance to center were evenly distributed; most patients had government (n = 2923 [46.9%]) or private insurance (n = 2858 [45.8%]), and were treated at a comprehensive community center (n = 2423 [38.9%]) or an academic center (n = 1905 [30.6%]) (Table 1).

**Table 1:** Demographic and Clinical Characteristics of Patients with HR+/HER2+ Metastatic Breast Cancer.

	ALL	Treatment		
	n (%)	Chemotherapy n (%)	Hormonal Therapy n (%)	*P*
	6234 (100.0)	2464 (39.5)	3770 (60.5)	
Age, years				
<50	1632 (26.2)	740 (30.0)	892 (23.7)	<0.001
50–70	3425 (54.9)	1403 (56.9)	2022 (53.6)	
>70	1177 (18.9)	321 (13.0)	856 (22.7)	
Sex				
Female	6141 (98.5)	2427 (98.5)	3714 (98.5)	1.00
Male	93 (1.5)	37 (1.5)	56 (1.5)	
Race				
White	4491 (72.0)	1667 (67.7)	2824 (74.9)	<0.001
African American	1054 (16.9)	486 (19.7)	568 (15.1)	
Asian	195 (3.1)	80 (3.2)	115 (3.1)	
Hispanic/Latinos	395 (6.3)	194 (7.9)	201 (5.3)	
Others	65 (1.0)	28 (1.1)	37 (1.0)	
Unknown	34 (0.5)	9 (0.4)	25 (0.7)	
Grade				
Grade 1/Grade 2	2192 (35.2)	779 (31.6)	1413 (37.5)	<0.001
Grade 3/Grade 4	2912 (46.7)	1216 (49.4)	1696 (45.0)	
Unknown	1130 (18.1)	469 (19.0)	661 (17.5)	
Visceral Involvement				
Yes	3276 (52.6)	1462 (59.3)	1814 (48.1)	<0.001
No	2958 (47.4)	1002 (40.7)	1956 (51.9)	
Number of Metastatic Sites				
1	4003 (64.2)	1485 (60.3)	2518 (66.8)	<0.001
2–3	2148 (34.5)	944 (38.3)	1204 (31.9)	
>3	83 (1.3)	35 (1.4)	48 (1.3)	
Charlson-Deyo Comorbidity Score				
0	5161 (82.8)	2088 (84.7)	3073 (81.5)	0.007
1	833 (13.4)	297 (12.1)	536 (14.2)	
2	158 (2.5)	49 (2.0)	109 (2.9)	
≥3	82 (1.3)	30 (1.2)	52 (1.4)	
Year of Diagnosis				
2010	863 (13.8)	321 (13.0)	542 (14.4)	<0.001
2011	905 (14.5)	316 (12.8)	589 (15.6)	
2012	1054 (16.9)	386 (15.7)	668 (17.7)	
2013	1059 (16.9)	468 (19.0)	591 (15.7)	
2014	1148 (18.4)	462 (18.8)	686 (18.2)	
2015	1205 (19.3)	511 (20.7)	694 (18.4)	
Insurance				
Government	2923 (46.9)	1068 (43.3)	1855 (49.2)	<0.001
Not Insured	354 (5.7)	137 (5.6)	217 (5.8)	
Private	2858 (45.8)	1211 (49.1)	1647 (43.7)	
Unknown	99 (1.6)	48 (1.9)	51 (1.4)	
Community Median Income, $				
<38,000	1123 (18.0)	479 (19.4)	644 (17.1)	0.05
38,000–62,999	2974 (47.7)	1161 (47.1)	1813 (48.1)	
≥63,000	2115 (33.9)	819 (33.2)	1296 (34.4)	
Unknown	22 (0.4)	5 (0.2)	17 (0.5)	
Center Type				
Community Cancer Center	629 (10.1)	249 (10.1)	380 (10.1)	0.17
Comprehensive Community Center	2423 (38.9)	919 (37.3)	1504 (39.9)	
Academic Center	1905 (30.5)	776 (31.5)	1129 (29.9)	
Integrated Network	1277 (20.5)	520 (21.1)	757 (20.1)	
Distance to Center, Miles				
<4	1498 (24.0)	586 (23.8)	912 (24.2)	0.69
4–8.9	1626 (26.1)	630 (25.6)	996 (26.4)	
9–17.9	1441 (23.1)	569 (23.1)	872 (23.1)	
≥18	1669 (26.8)	679 (27.6)	990 (26.3)	
anti-HER2				
Yes	2646 (42.4)	1175 (47.7)	1471 (39.0)	<0.001
No	3569 (57.3)	1280 (51.9)	2289 (60.7)	
Unknown	19 (0.3)	9 (0.4)	10 (0.3)	

Abbreviation: P, p-value.

### Treatment utilization

First-line treatment data revealed hormonal therapy (n = 3770 [60.5%]) was more commonly utilized in the first-line setting for MBC than chemotherapy (n = 2464 [39.5%]) and less than half of all patients received anti-HER2 therapy (n = 2646 [42.4%]). Distribution of treatment utilization did not meaningfully vary by year. Median follow-up of alive patients was similar for patients receiving hormonal therapy (33.8 months, IQR: 22.1–48.2) vs. chemotherapy (34.3 months, IQR: 23.6–46.7). Characteristics between treatment groups were significantly imbalanced: older white patients, with grade 1/grade 2 disease, and comorbidity scores ≥1 or ≥2 were more likely to receive hormonal therapy; while chemotherapy was more common among Hispanic/Latinos and African Americans, those with visceral involvement, >1 metastatic site, patients with private insurance, and those diagnosed in 2013. The receipt of anti-HER2 therapy was also significantly different between groups, patients treated with chemotherapy were more likely to receive combination therapy than those treated with hormonal therapy (47.7% vs. 39.0% p < 0.001) (Table 1). Multivariable logistic regression results revealed similar results, with the exception of anti-HER2, insurance, and number of metastatic sites, each of which were not significant predictors of treatment receipt (Table 2).

**Table 2:** Factors Associated with Hormonal Treatment Receipt.

Variable	OR	95% CI	*P*
Age, years			
<50	Reference		
50–70	1.02	0.87–1.18	0.83
>70	1.87	1.52–2.30	<0.001
Grade			
Grade 3/Grade 4	Reference		
Grade 1/Grade 2	1.25	1.09–1.42	<0.001
Visceral Involvement			
No	Reference		
Yes	0.64	0.56–0.72	<0.001
Charlson-Deyo comorbidity score			
0	Reference		
1	1.27	1.04–1.55	0.02
2	1.50	0.97–2.36	0.07
≥3	1.45	0.77–2.85	0.26
Race			
White	Reference		
African American	0.74	0.62–0.88	0.001
Asian	0.89	0.62–1.30	0.54
Hispanic/Latinos	0.66	0.51–0.86	0.002
Others	0.68	0.36–1.29	0.23
Year of Diagnosis			
2010	Reference		
2011	1.17	0.94–1.45	0.16
2012	1.08	0.87–1.33	0.49
2013	0.77	0.62–0.95	0.01
2014	0.87	0.71–1.07	0.18

Abbreviation: OR, odds ratio; P, p-value.

### Outcomes

**Figure 2:**
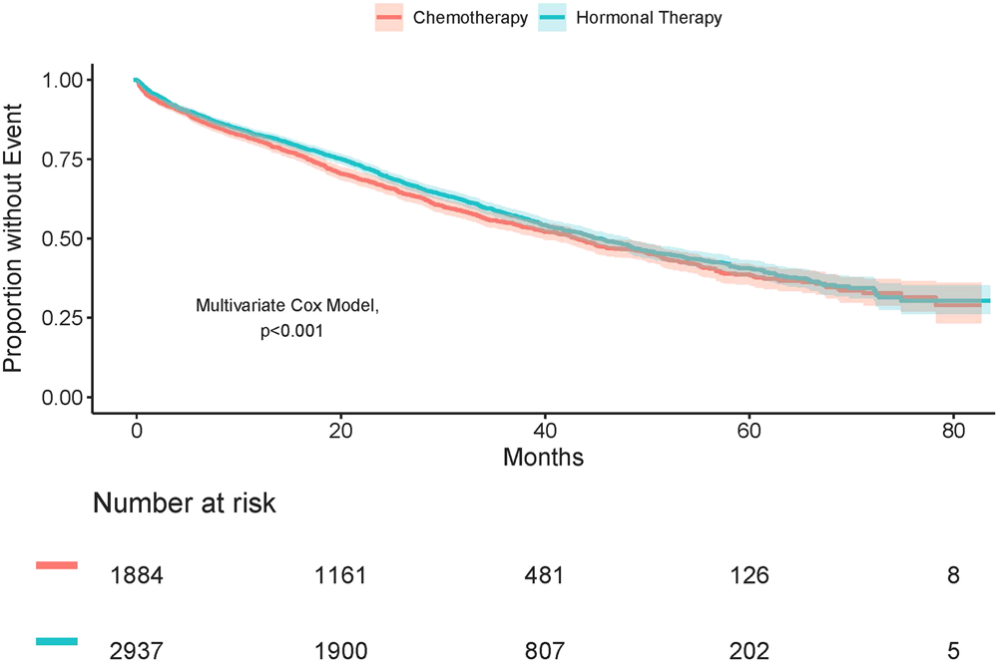
Overall survival among HR+/HER2+ metastatic breast cancer patients treated with chemotherapy or hormonal therapy. HR, hormone receptor; HER, human epidermal growth factor.

Median OS for all patients was 44.4 months (95% CI, 42.5–46.5) and the 5-year OS rate was 40.0% (95% CI: 37.8–41.9). Univariate analysis revealed there was no difference in 5-year OS between the hormonal therapy and chemotherapy treatment groups (40.6% vs. 38.6% p = 0.05). However, multivariate analysis indicated improved survival was independently associated with receiving hormonal therapy (HR: 0.84; 95% CI, 0.76–0.92, p < 0.001) (Fig. 2). Additional prognostic factors for improved survival included: younger age, and grade 1/grade 2 disease. Whereas, African Americans and those with at least 1 comorbidity or visceral involvement exhibited higher death rates (Table 3, Table A1).

**Table 3:** Multivariate Overall Survival Analysis.

Variable	HR	95% CI	*P*
Treatment Group			
Chemotherapy	Reference		
Hormonal therapy	0.84	0.76–0.92	<0.001
Anti-HER2 Therapy			
No	Reference		
Yes	0.66	0.57–0.77	<0.001
Age, years			
<50	Reference		
50–70	1.72	1.51–1.95	<0.001
>70	2.95	2.54–3.42	<0.001
Race			
White	Reference		
African American	1.37	1.21–1.54	<0.001
Asian	0.97	0.72–1.33	0.87
Hispanic/Latinos	0.87	0.70–1.09	0.22
Others	0.63	0.35–1.13	0.12
Grade			
Grade 3/Grade 4	Reference		
Grade 1/Grade 2	0.90	0.82–0.99	0.03
Visceral Involvement			
No	Reference		
Yes	1.43	1.30–1.57	<0.001
Year of Diagnosis			
2010	Reference		
2011	1.02	0.88–1.17	0.82
2012	0.90	0.78–1.05	0.17
2013	0.98	0.82–1.17	0.79
2014	1.31	1.08–1.58	0.005
Charlson-Deyo comorbidity score			
0	Reference		
1	1.29	1.13–1.47	<0.001
2	1.74	1.36–2.22	<0.001
≥3	2.00	1.40–2.87	<0.001

Abbreviation: HR, hazard ratio; P, p-value.

**Figure 3:**
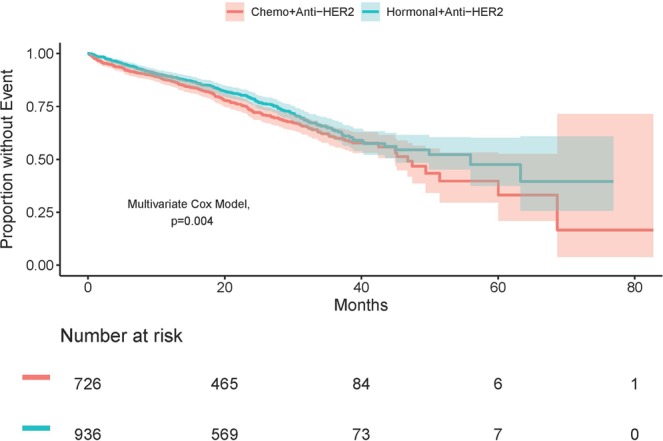
Overall survival among HR+/HER2+ metastatic breast cancer patients treated with chemotherapy + anti-HER2 therapy or hormonal therapy + anit-HER2 therapy. Chemo, chemotherapy; HR, hormone receptor; HER, human epidermal growth factor.

Overall, patients treated with combination therapy (chemotherapy or hormonal therapy + anti-HER2 therapy) had lower death rates than those treated with monotherapy; of those treated with combination therapy, the hormonal group reported the highest 5-year survival rates (hormonal + anti-HER2: 47.5% vs. chemotherapy + anti-HER2: 39.8% vs. hormonal: 38.5% vs. chemotherapy: 36.3% p < 0.001). After controlling for prognostic factors, multivariate subgroup analysis of patients treated with targeted therapy supported the univariate results: patients receiving hormonal therapy combined with anti-HER2 therapy experienced a lower rate of death than those treated with chemotherapy and anti-HER2 therapy (HR: 0.74; 95% CI 0.61–0.91, p = 0.004) (Table 4, Table A2) (Fig. 3). Furthermore, improved survival within this subgroup of patients was associated with younger age, whereas race (African American vs. white) and having 3 or more comorbidities, and visceral involvement were predictive of diminished overall survival (Table 4).

**Table 4:** Multivariate Overall Survival Analysis (Anti-Her2 Subgroup).

Variable	HR	95% CI	*P*
Treatment Group			
Chemotherapy + Anti-HER2	Reference		
Hormonal therapy + Anti-HER2	0.74	0.61–0.91	0.004
Age, years			
<50	Reference		
50–70	1.67	1.29–2.14	<0.001
>70	2.70	1.99–3.67	<0.001
Race			
White	Reference		
African American	1.38	1.07–1.79	0.01
Asian	1.33	0.77–2.29	0.30
Hispanic/Latinos	0.82	0.54–1.25	0.36
Others	0.86	0.35–2.10	0.74
Grade			
Grade 3/Grade 4	Reference		
Grade 1/Grade 2	0.92	0.75–1.12	0.39
Visceral Involvement			
No	Reference		
Yes	1.50	1.22–1.84	<0.001
Year of Diagnosis			
2010	Reference		
2011	1.16	0.39–3.44	0.79
2012	1.71	0.64–4.56	0.29
2013	1.68	0.65–4.38	0.29
2014	2.12	0.81–5.55	0.13
Charlson-Deyo comorbidity score			
0	Reference		
1	1.16	0.88–1.54	0.28
2	1.19	0.65–2.19	0.58
≥3	4.14	1.93–8.89	<0.001

Abbreviation: HR, hazard ratio; P, p-value.

## Discussion

Real-world treatment utilization patterns for patients diagnosed with ER+/PR+/HER2+ stage IV breast cancer are not well established. While combining chemotherapy with anti-HER2 therapy remains a standard approach for HER2-positive breast cancer, there continues to be a debate regarding the best first-line treatment approach in the metastatic setting. In this analysis of 6,000+ real-world metastatic ER+ or PR+ and HER2+ breast cancer patients, we identified two distinct first-line treatment patterns: hormonal therapy and chemotherapy. The majority of patients (60%) received hormonal therapy, the receipt of which was associated with older age, grade 1/grade 2 disease, no visceral involvement, and higher comorbidity scores. Overall, less than half of the analyzed patients received anti-HER2 therapy (42%). The proportion of patients treated with anti-HER2 therapy was slightly more pronounced within the chemotherapy group and was significantly greater than those who received anti-HER2 plus hormonal therapy (48% vs. 39%, p < 0.001). These results are concerning, given the extent of evidence in support of anti-HER2 therapy^3,4^ and the majority of HR+/HER2+ metastatic breast cancer patients included in the registHER cohort received tratuzumab^13^. A possible explanation may be the lack of specific treatment guidelines, which will require prospective randomized studies demonstrating the first-line combination of hormonal therapy and anti-Her2 therapy is superior to chemotherapy and anti-HER2 in metastatic HR+/HER+ breast cancer patients.

Although standard of care for triple positive breast cancer patients (ER+/PR+/HER2+) typically is chemotherapy plus anti-HER2 therapy, this study’s findings suggest, in the metastatic setting, hormonal therapy is the more common care path, and, when combined with anti-HER2 therapy, may provide the best survival outcomes. Furthermore, specific patient attributes were associated with hormonal treatment, indicating clinicians are more likely to prescribe this therapy to patients who may not be fit to receive high-intensity chemotherapy. These findings validate and extend the clinical outcomes of a previous study^13^, which reported improved overall survival and progression free survival associated with dual targeting HR+and HER2+, with or without chemotherapy. Importantly, our study confirms the benefit of targeted therapy among a larger sample of patients (6234 vs. 530) diagnosed more recently (2010–2015 vs. 2003–2006), suggesting chemotherapy may not be the ideal care path, as improved overall survival was associated with de-escalated treatment regimens. Furthermore, our treatment pattern data suggest temporal changes in treatment utilization may have occurred, perhaps in response to the outcomes reported in the registHER study^13^, as hormonal therapy was more common than chemotherapy and targeted therapy, which has been previously reported as the favored treatment pattern among this subpopulation of breast cancer patients^13^. These results elucidate, the preferred and potentially more effective real-world first-line approach in HR+/HER2+ breast cancer is hormonal treatment, particularly for those patients who have diminished physiological function, grade 1/grade 2 disease, and no visceral involvement. This may be extended to support a similar approach within the metastatic HR+/HER2-negative breast cancer population, recommending delaying chemotherapy until the benefit of hormonal therapy lessens, tumor becomes refractory to hormonal therapy, and/or a visceral crises is imminent^8^.

Our survival analysis revealed the less intensive treatment pattern was likely the best option for this patient population, as the cohort treated with hormonal therapy had better survival than those treated with chemotherapy. Furthermore, death rates were lower among patients receiving anti-HER2 therapy, regardless if they received chemotherapy or hormonal therapy (5-year OS: 44% (anit-HER2) vs. 38% (no anti-HER2), p < 0.001). Patients administered anti-HER2 therapy in addition to hormonal treatment exhibited evidence of further improvements in survival when compared to chemotherapy plus anti-HER2 therapy (5-year OS: 48% vs. 40%, p = 0.002).

Several large-scale studies^3,4^ have supported trastuzumab, an anti-HER2 targeted therapy, in combination with hormonal therapies for postmenopausal women with ER+/HER2+ metastatic breast cancer. The benefits offered by trastuzumab are also apparent when it is used either sequential to or concurrently with adjuvant or neo-adjuvant chemotherapy regimens^14^ for ER+/HER2+ breast tumors. Our findings support the consideration of hormonal therapy plus anti-HER2 therapy in the real-world setting, indicating this may be preferred over chemotherapy for this subgroup of patients.

More recently, dual targeting of HER2 has proven to be an effective strategy for increasing tumor shrinkage compared with single-agent HER2 therapy, with or without chemotherapy. The addition of pertuzumab to trastuzumab and chemotherapy resulted in a survival benefit leading this dual HER2 blockade to be considered a standard of care as first-line therapy for advanced HER2+ disease^15,16^. Furthermore, data from two dual HER2 targeting studies^17,18^ report outcomes that suggest a potent cocktail of drugs that more completely block the HER network causes pathologic complete response in a subset of patients with ER+/HER2+ tumors, without using chemotherapy.

These studies suggest the future treatment paradigm for HR+/HER2+ breast cancer patients may shift towards chemotherapy sparing regimens. Our results from this NCDB population of HR+/HER2+ breast cancer patients suggest clinicians are already making this transition in the metastatic setting. As a result, the patients who receive hormonal therapy have lower death rates than those treated with chemotherapy, after adjustment for clinically relevant prognostic factors. On the other hand, our findings suggest, anti-HER2 therapies, although indicated for this patient population, may be underutilized; only 42% received targeted therapy, even though all patients were HER2+. Although the dual HER2 treatment regimens were not explored in this analysis, our findings suggest that survival benefit may be associated with anti-HER2 targeted therapy, particularly within the hormonal therapy plus anti-HER2 treatment pattern. Notably, the use of anti-HER2 therapy was most common within the chemotherapy group, suggesting clinicians are still more likely to pair anti-HER2 therapy with chemotherapy than hormonal therapy. Thus, future studies exploring the combination of hormonal therapy with dual anti-Her2 treatment would be desirable in this setting.

In addition to these real-world first-line treatment patterns, we also identified significant prognostic factors for survival. After controlling for treatment receipt, survival benefit was independently also associated with younger age and fewer comorbidities, whereas African Americans (compared to whites) and those with visceral involvement had higher death rates, despite which treatment they received. These results, for the first time, reveal individual-level characteristics associated with overall survival within a real-world population of HR+/HER+ metastatic breast cancer patients treated with first-line chemotherapy or hormonal therapy. The association of age and number of comorbidities with survival outcomes are not surprising; however, the identified racial disparity may not be biologically explained, warranting further investigation.

This study had several limitations. The NCDB is a hospital-based registry, therefore, it is not representative of the general population; furthermore, because the treatment data is collected from multiple institutions we were unable to account for center-specific treatment protocols. We also assumed differences in overall survival were primarily attributable to disease; however, because the NCDB does not include cause of death, survival differences may have been associated with patient-level variables not included in the database. Follow up time may also have been limited, given the cohort did not include patients diagnosed prior to 2010. However, because HER2+ status was not consistently reported prior to 2010, the cohort could not be reliably constructed if patients diagnosed prior to 2010 were included. Additional patient follow-up extending beyond 1745 deaths and median follow-up of 34 months, will result in more precise statistical estimates. Furthermore, several patients were excluded from the analysis due to: 1) incomplete survival data, the majority of which were diagnosed in 2015 and 2) missing grade of disease. Treatment information was also limited, individual agents were not reported, which restricted our ability to compare outcomes based upon agent received (i.e. dual HER2 blockade). The NCDB also does not include adverse events or quality-of-life measures; thus, we were unable to evaluate if safety and/or quality-of-life outcomes differed between those treated with hormonal therapy vs. chemotherapy. As appropriate for retrospective analyses of observational studies, statistical conclusions identify associations among treatment cohorts but avoid causality for which statistical inference may require randomized study.

## Conclusions

We conducted a multi-center retrospective cohort analysis of HR+/HER2+ stage IV breast cancer patients, explicitly evaluating treatment utilization and overall survival among patients treated with hormonal therapy or chemotherapy. Our results reveal the currently preferred and practiced treatment approaches are consistent with the general trend of de-escalation of therapy, which avoids chemotherapy unless strongly indicated. Furthermore, we report the preferred hormonal treatment pattern is associated with improved survival compared to chemotherapy, with an increase in survival benefit after the addition of anti-HER therapy. These findings support the consideration of chemotherapy sparing regimens with anti-HER2 therapy plus hormonal therapy as a viable and effective treatment option for the first –line treatment of patients with HR+/HER2+ metastatic breast cancer. Dual HER2 blockade with lapatinib or pertuzumab and trastuzumab along with hormonal therapy is likely to confer added benefit compared to trastuzumab and hormonal therapy alone, suggesting future trials may focus on evaluating the toxicity burden of dual HER2 blockade plus hormonal therapy vs. chemotherapy. Randomized studies evaluating combination therapy, while aiming to identify relevant treatment biomarkers, will likely improve outcomes within this subgroup of breast cancer patients.

## Supplementary information

Appendix

## Data Availability

The data that support the findings of this study are available from the American College of Surgeons but restrictions apply to the availability of these data, which were used under license for the current study, and so are not publicly available.
