# Supplementary material for: Real-world Treatment Patterns and Outcomes in HR+/HER2+ Metastatic Breast Cancer Patients: A National Cancer Database Analysis
Source: Sci Rep. 2019 Dec 2;9:18126. doi: 10.1038/s41598-019-54402-9 (PMC6889133; doi:10.1038/s41598-019-54402-9)
Supplement: Supplementary file 1 — Appendix [file 41598_2019_54402_MOESM1_ESM.docx]

**Real-world Treatment Patterns and Outcomes in HR+/HER2+ Metastatic Breast Cancer Patients: A National Cancer Database Analysis**

Abby B. Statler, Brian P. Hobbs, Wei Wei, Annie Gupta, Cassann N. Blake, and Zeina A. Nahleh

**Appendix**

**Figure A1**

**
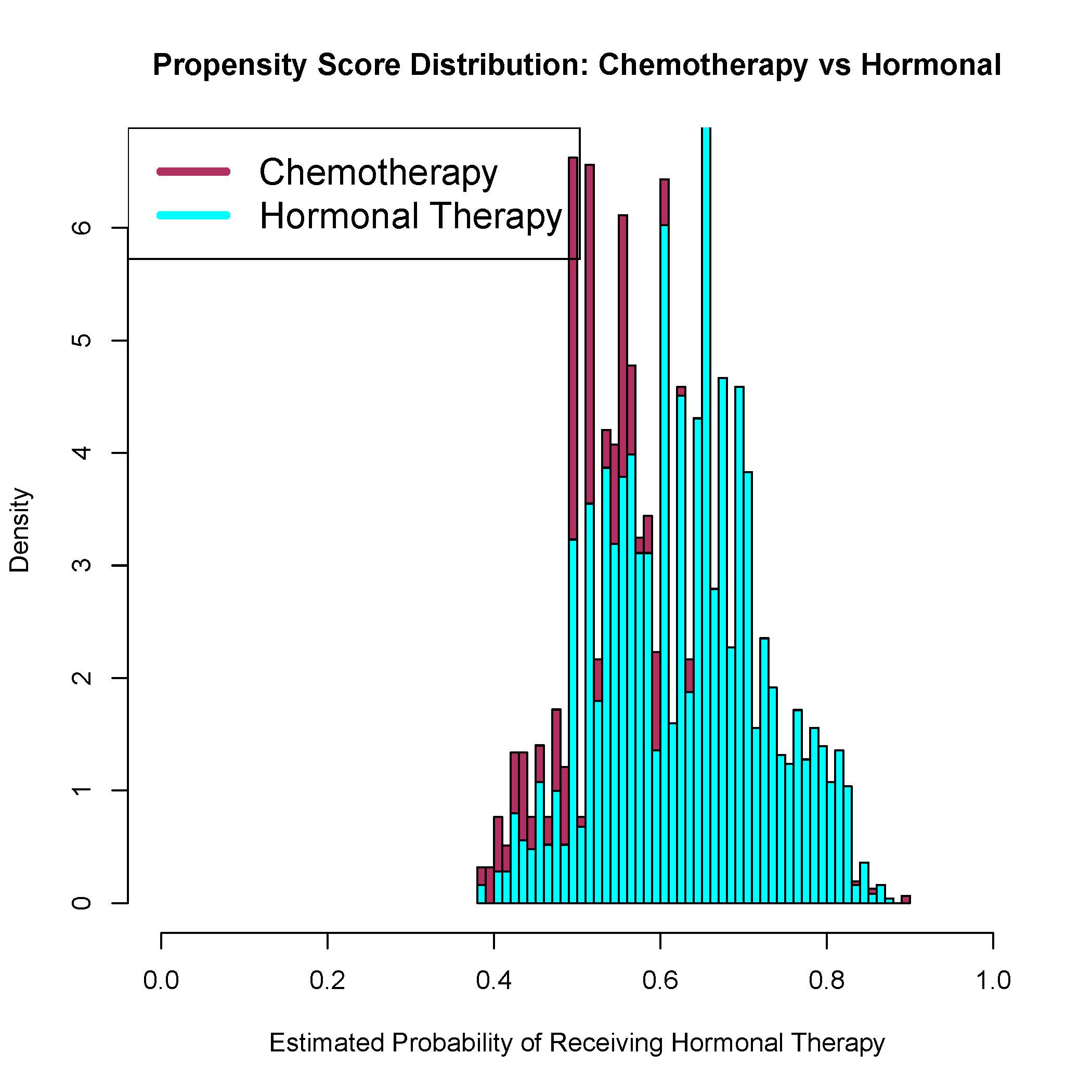
**

**Figure A2**

**
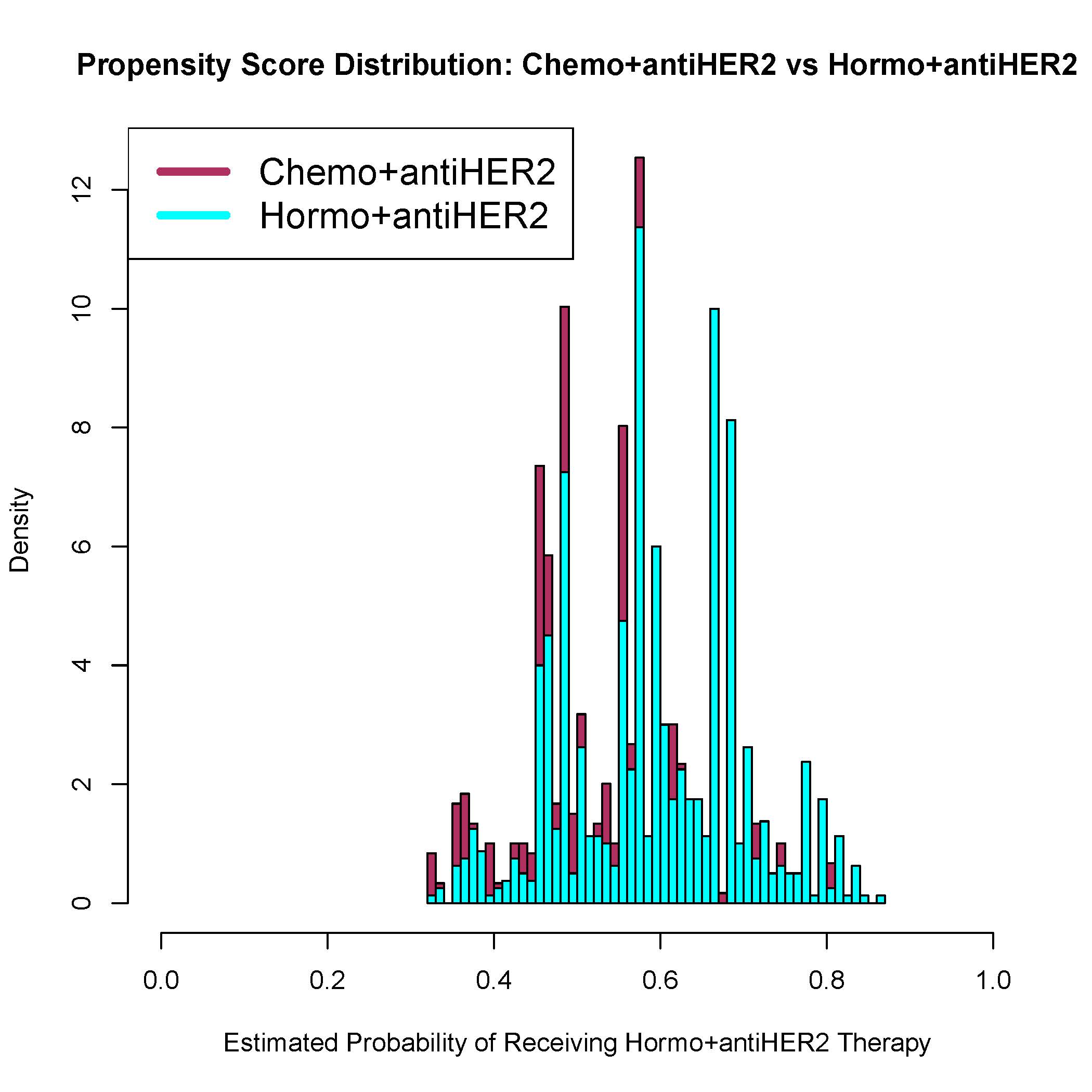
**

| Table A1. Multivariate Overall Survival Analysis | |  |  |
| --- | --- | --- | --- |
| **Variable** | **HR*** | **95% CI** | ***P*** |
| **Treatment Group** |  |  |  |
| Chemotherapy | Reference |  |  |
| Hormonal therapy | 0.84 | 0.76 - 0.92 | <0.001 |
| **Anti-HER2 Therapy** |  |  |  |
| No | Reference |  |  |
| Yes | 0.66 | 0.57 - 0.77 | <0.001 |
| **Age, years** |  |  |  |
| <50 | Reference |  |  |
| 50-70 | 1.72 | 1.51 - 1.96 | <0.001 |
| >70 | 3.12 | 1.41 - 6.90 | 0.005 |
| **Race** |  |  |  |
| White | Reference |  |  |
| African American | 1.33 | 0.87 - 2.03 | 0.19 |
| Asian | 0.96 | 0.68 - 1.36 | 0.83 |
| Hispanic/Latinos | 0.84 | 0.45 - 1.55 | 0.57 |
| Others | 0.60 | 0.26 - 1.39 | 0.23 |
| **Grade** |  |  |  |
| Grade 3 / Grade 4 | Reference |  |  |
| Grade 1 / Grade 2 | 0.92 | 0.68 - 1.25 | 0.59 |
| **Visceral Involvement** |  |  |  |
| No | Reference |  |  |
| Yes | 1.37 | 0.76 - 2.47 | 0.3 |
| **Year of Diagnosis** |  |  |  |
| 2010 | Reference |  |  |
| 2011 | 1.01 | 0.86 - 1.19 | 0.91 |
| 2012 | 0.89 | 0.71 - 1.12 | 0.33 |
| 2013 | 0.96 | 0.69 - 1.33 | 0.79 |
| 2014 | 1.28 | 0.85 - 1.92 | 0.25 |
| **Charlson-Deyo comorbidity score** |  |  |  |
| 0 | Reference |  |  |
| 1 | 1.31 | 0.99 - 1.73 | 0.06 |
| 2 | 1.79 | 1.08 - 2.98 | 0.02 |
| ≥3 | 2.10 | 0.99 - 4.47 | 0.05 |
| Abbreviation: HR, hazard ratio; P, p-value |  |  |  |
| *Additionally adjusted for the propensity of hormonal vs. chemotherapy receipt | | |  |

| Table A2. Multivariate Overall Survival Analysis (Anti-Her2 Subgroup) | | |  |
| --- | --- | --- | --- |
| **Variable** | **HR*** | **95% CI** | ***P*** |
| **Treatment Group** |  |  |  |
| Chemotherapy + Anti-HER2 | Reference |  |  |
| Hormonal therapy + Anti-HER2 | 0.74 | 0.61 - 0.91 | 0.004 |
| **Age, years** |  |  |  |
| <50 | Reference |  |  |
| 50-70 | 1.65 | 1.27 - 2.15 | 0.0002 |
| >70 | 3.37 | 0.63 - 17.95 | 0.16 |
| **Race** |  |  |  |
| White | Reference |  |  |
| African American | 1.18 | 0.34 - 4.07 | 0.80 |
| Asian | 1.19 | 0.45 - 3.18 | 0.72 |
| Hispanic/Latinos | 0.65 | 0.11 - 3.86 | 0.64 |
| Others | 0.68 | 0.09 - 5.07 | 0.70 |
| **Grade** |  |  |  |
| Grade 3 / Grade 4 | Reference |  |  |
| Grade 1 / Grade 2 | 1.08 | 0.31 - 3.73 | 0.91 |
| **Visceral Involvement** |  |  |  |
| No | Reference |  |  |
| Yes | 1.24 | 0.29 - 5.28 | 0.78 |
| **Year of Diagnosis** |  |  |  |
| 2010 | Reference |  |  |
| 2011 | 1.13 | 0.37 - 3.41 | 0.83 |
| 2012 | 1.60 | 0.53 - 4.81 | 0.41 |
| 2013 | 1.51 | 0.44 - 5.24 | 0.51 |
| 2014 | 1.83 | 0.42 - 7.89 | 0.42 |
| **Charlson-Deyo comorbidity score** |  |  |  |
| 0 | Reference |  |  |
| 1 | 1.31 | 0.52 - 3.32 | 0.57 |
| 2 | 1.49 | 0.25 - 8.77 | 0.66 |
| ≥3 | 5.81 | 0.41 - 82.22 | 0.19 |
| Abbreviation: HR, hazard ratio; P, p-value | |  |  |
| *Additionally adjusted for the propensity of hormonal therapy + antiHER vs. chemotherapy + anti-HER | | |  |
